# The Burden of Type 2 Diabetes in Adolescents and Young Adults in China: A Secondary Analysis from the Global Burden of Disease Study 2021

**DOI:** 10.34133/hds.0210

**Published:** 2024-12-17

**Authors:** Junting Yang, Siwei Deng, Houyu Zhao, Feng Sun, Xiantong Zou, Linong Ji, Siyan Zhan

**Affiliations:** ^1^ Department of Epidemiology and Biostatistics, School of Public Health, Peking University, Beijing 100191, China.; ^2^ Center for Intelligent Public Health, Institute for Artificial Intelligence, Peking University, Beijing 100871, China.; ^3^ Key Laboratory of Epidemiology of Major Diseases (Peking University), Ministry of Education, Beijing 100191, China.; ^4^ School of Medicine, Chongqing University, Chongqing 400030, China.; ^5^ The Department of Endocrinology and Metabolism, Peking University People’s Hospital, Beijing 100044, China.; ^6^ Research Center of Clinical Epidemiology, Peking University Third Hospital, Beijing 100191, China.

## Abstract

**Background:** Early-onset type 2 diabetes (T2D) is an increasingly serious public health issue, particularly in China. This study aimed to analyze the characteristics of disease burden, secular trend, and attributable risk factors of early-onset T2D in China. **Methods:** Using data from the Global Burden of Disease (GBD) 2021, we analyzed the age-standardized rate (ASR) of incidence, disability-adjusted life years (DALYs), and mortality rates of T2D among individuals aged 15 to 39 years in China from 1990 to 2021. Joinpoint regression analysis was employed to analyze secular trend, calculating the average annual percent change (AAPC). We also examined changes in the proportion of early-onset T2D within the total T2D burden and its attributable risk factors. **Results:** From 1990 to 2021, the ASR of incidence of early-onset T2D in China increased from 140.20 [95% uncertainty interval (UI): 89.14 to 204.74] to 315.97 (95% UI: 226.75 to 417.55) per 100,000, with an AAPC of 2.67% (95% CI: 2.60% to 2.75%, *P* < 0.001). DALYs rose from 116.29 (95% UI: 78.51 to 167.05) to 267.47 (95% UI: 171.08 to 387.38) per 100,000, with an AAPC of 2.75% (95% CI: 2.64% to 2.87%, *P* < 0.001). Mortality rates slightly decreased from 0.30 (95% UI: 0.24 to 0.38) to 0.28 (95% UI: 0.23 to 0.34) per 100,000, with an AAPC of −0.22% (95% CI: −0.33% to −0.11%, *P* < 0.001). The 15 to 19 years age group showed the fastest increase in incidence (AAPC: 4.08%, 95% CI: 3.93% to 4.29%, *P* < 0.001). The burden was consistently higher and increased more rapidly among males compared to females. The proportion of early-onset T2D within the total T2D burden fluctuated but remained higher than global levels. In 2021, high body mass index (BMI) was the primary attributable risk factor for DALYs of early-onset T2D (59.85%, 95% UI: 33.54% to 76.65%), and its contribution increased substantially from 40.08% (95% UI: 20.71% to 55.79%) in 1990, followed by ambient particulate matter pollution (14.77%, 95% UI: 8.24% to 21.24%) and diet high in red meat (9.33%, 95% UI: −1.42% to 20.06%). **Conclusion:** The disease burden of early-onset T2D in China is rapidly increasing, particularly among younger populations and males. Despite a slight decrease in mortality rates, the continued rapid increase in incidence and DALYs indicates a need for strengthened prevention and management strategies, especially interventions targeting younger age groups. High BMI and environmental pollution emerge as primary risk factors and should be prioritized in future interventions.

## Introduction

Type 2 diabetes (T2D) is a serious public health threat. In 2021, it was estimated that globally 537 million adults aged 20 to 79 years were living with diabetes, causing 6.7 million deaths and at least 966 billion dollars in health expenditure [1]. As a progressive metabolic disease, diabetes has long been thought to mainly affect middle-aged and elderly populations. However, the incidence of early-onset T2D, defined as T2D diagnosed before the age of 40, has risen rapidly. A study utilizing Global Burden of Disease (GBD) 2019 data found that the age-standardized rates (ASRs) of T2D incidence for individuals aged 15 to 39 years increased from 117.2 per 100,000 in 1990 to 183.4 per 100,000 in 2019 [2]. Additionally, studies from countries such as the United States [3] and the United Kingdom [4] have also reported a gradual increase in incidence rates, drawing considerable attention from researchers [5–8].

Individuals with early-onset T2D are exposed to hyperglycemia for a longer duration, and the disease progression, including the development of insulin resistance and β cell dysfunction, is more rapid compared to late-onset patients [9,10]. Consequently, these patients often struggle with poorer glycemic control and higher risks of complications [11–13]. Studies have reported increased risks of cardiovascular, neurological, and renal complications in early-onset T2D patients [14–18]. Moreover, research indicates that commonly used oral glucose-lowering drugs (OGLDs) are less effective for early-onset T2D patients, leading to higher rates of treatment failure [19,20].

In China, early-onset T2D has emerged as a growing public health issue. A previous study has reported the secular trend and distribution characteristics of the disease burden of early-onset T2D globally from 1990 to 2019, revealing large regional differences and a higher burden in Asia [8]. However, this study did not conduct an in-depth analysis of the situation in China. As one of the most populous countries in Asia, China has experienced a high and rapidly growing prevalence of early-onset T2D [21–23]. The 2010 National Survey in China showed that the prevalence of T2D was 4.5% among 18 to 29 years and 6.6% among 30 to 39 years, while the prevalence of prediabetes was 40 to 50% among those under 40 years old [22]. Another study showed that the overall prevalence of diabetes in mainland China increased from 0.06% in 1979 to 7.09% in 2011 [23]. Despite these alarming trends, there has been a lack of systematic analysis regarding the disease burden and attributable risk factors of early-onset T2D specific to China, underscoring the urgent need for comprehensive research in this area.

Therefore, the aim of this study is to use data from the GBD 2021 to analyze the population distribution characteristics, the secular trends, and the attributable risk factors of the disease burden of early-onset T2D in China.

## Methods

### Data sources

Data on the disease burden of T2D and its attributable risk factors for both China and global populations were extracted from the GBD 2021 study. The GBD collaborators conducted comprehensive and systematic assessments of incidence, disability-adjusted life years (DALYs), and mortality for 369 diseases and injuries, as well as comparative risks for 88 risk factors, across 204 countries and territories between 1990 January 1 and 2021 December 31. These data sources included censuses, household surveys, civil registration and vital statistics, disease registries, health service utilization, sensors/monitors, satellite imaging, and others. For China, the diabetes data in the GBD study were specifically derived from a range of national surveys, cohort studies, and epidemiological surveillance systems, such as the China Disease Surveillance Points system, the China Chronic Disease and Risk Factor Surveillance, and various urban and rural cohort studies [24]. The detailed methods of GBD 2021 are reported in prior studies [2]. While early-onset T2D is commonly defined as T2D diagnosed before the age of 40 [5,7], the GBD data include information only for populations aged 15 years and above. Therefore, our study specifically analyzed T2D patients aged 15 to 39 years and extracted data for all T2D patients across the entire population to examine the changes in the proportion of early-onset T2D patients within the overall T2D population. Additionally, we extracted age- and sex-specific data on incidence, DALYs, and mortality rates for early-onset T2D, as well as data on DALYs attributable to various risk factors, along with their corresponding 95% uncertainty intervals (UIs). The 95% UIs for every metric in the GBD study were determined by calculating the 25th and 975th percentiles from 1000 draws of the posterior distribution. All data were accessed through the Global Health Data Exchange (https://ghdx.healthdata.org/gbd-results-tool).

### Estimation of disease burden of T2D

In GBD 2021, diabetes was defined as a fasting plasma glucose ≥126 mg/dl (7 mmol/L) or reported treatment for diabetes. However, in the GBD datasets, only 20% of data sources had information on diabetes type, and diagnostic criteria for classifying diabetes types were inconsistent across different sources [24]. Therefore, in the GBD study, T2D was estimated indirectly by subtracting the number of cases definitively classified as type 1 diabetes from the total number of diabetes cases. A Bayesian meta-regression tool, named DisMod MR-2.1, was used to estimate nonfatal burdens of diabetes, while fatal burdens of diabetes were estimated using the Cause of Death Ensemble model in GBD 2019 [8]. In this study, we measured the burden of early-onset T2D using incidence, DALYs, and mortality. DALYs of early-onset T2D equal the sum of years lived with disability due to early-onset T2D and years of life lost due to premature death from early-onset T2D. The detailed estimation methods for these metrics in the GBD 2021 study have been extensively introduced in previous studies [2].

### Estimation of attributable risk factors for T2D

Our study used the proportional DALYs attributable to various risk factors as the metric to measure the contributions of these factors to early-onset T2D. This metric was calculated by dividing the attributable DALYs for each risk factor by the total DALYs for early-onset T2D. Attributable DALYs represent the potential reduction in disease burden if the risk factor were eliminated at the population level. It can be quantified using meta-analyses following the comparative risk assessment approach. More detailed calculation methods can be found in previously published literature [8,24,25]. In this analysis, alcohol use, which has controversial effects on T2D, and high fasting plasma glucose levels, which were assumed to contribute 100% to T2D in GBD 2021 [8,26], were excluded. Ultimately, 15 risk factors were analyzed, including diets low in fruit, vegetables, whole grains, and fiber; diets high in red meat, processed meat, and sugar-sweetened beverages; low physical activity; smoking; secondhand smoke; low temperature; high temperature; household air pollution from solid fuels; ambient particulate matter pollution; and high BMI. Relevant published literature offers comprehensive details on the definitions of risk factors and the specific estimation methodologies employed in the GBD Study 2021 [2].

### Statistical analysis

The ASR and corresponding 95% UIs of incidence, DALYs, and mortality for early-onset T2D were calculated based on the World Health Organization (WHO) standard population [27]. Rates were estimated per 100,000 population. To analyze secular trend in early-onset T2D burden, we employed joinpoint regression analysis, which segments the data and fits linear regression models within each segment to accurately capture shifts in trends. We calculated the average annual percent change (AAPC) and corresponding 95% confidence intervals (CIs) to quantify the rate of change [28]. The equation for the regression line is y=α+βx+ϵ, where $y=\ln\left(\mathrm{ASR}\right)$ and x is the calendar year. The AAPC is calculated using the formula $100\times \left({\text{exp}}^{\left(\beta \right)}-1\right)$, where positive values indicate an increasing trend and negative values indicate a decreasing trend.

To analyze the changing composition of early-onset T2D patients within the total T2D population from 1990 to 2021, we calculated the proportion of early-onset T2D burden (incidence, DALYs, and mortality) relative to the total T2D burden for each year. To assess the population distribution characteristics, we compared the incidence, DALYs, and mortality rates of early-onset T2D across sex and age groups.

Statistical analyses were performed using R software (version 4.2.3) for data preprocessing, descriptive statistics, and visualization. Joinpoint regression analyses were conducted using the Joinpoint Regression Program (version 5.2.0), utilizing the simplest model permitted by the data, and the optimal model was employed to determine the AAPC. The Monte Carlo permutation method was used to test the significance of change points in the joinpoint regression. All statistical tests were 2-sided, and a *P* < 0.05 was considered statistically significant.

## Results

### Secular trend of disease burden for early-onset T2D in China from 1990 to 2021

**Fig. 1.**
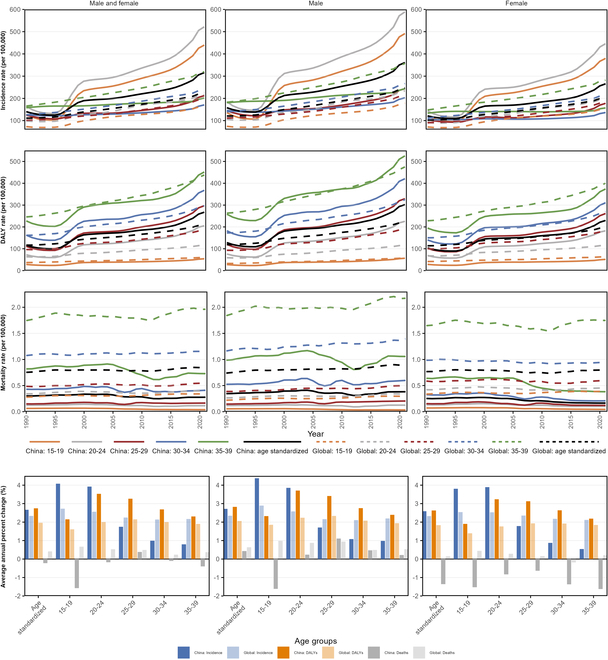
Secular trends of disease burden for early-onset T2D in China and globally by age groups and sex from 1990 to 2021.

From 1990 to 2021, China experienced a substantial increase in the age-standardized incidence, disability-adjusted life years (DALYs), and mortality rates for early-onset T2D. The ASR of incidence rate in China rose from 140.20 (95% UI: 89.14 to 204.74) per 100,000 in 1990 to 315.97 (95% UI: 226.75 to 417.55) per 100,000 in 2021, with an AAPC of 2.67% (95% CI: 2.60% to 2.75%, *P* < 0.001). The ASR of DALYs also increased from 116.29 (95% UI: 78.51 to 167.05) per 100,000 in 1990 to 267.47 (95% UI: 171.08 to 387.38) per 100,000 in 2021, with an AAPC of 2.75% (95% CI: 2.64% to 2.87%, *P* < 0.001). Mortality rates in China showed a slight decrease, from 0.30 (95% UI: 0.24 to 0.38) per 100,000 in 1990 to 0.28 (95% UI: 0.23 to 0.34) per 100,000 in 2021, with an AAPC of −0.22% (95% CI: −0.33% to −0.11%, *P* < 0.001). In comparison, global trends showed slower increases in incidence (AAPC: 2.34%, 95% CI: 2.32% to 2.37%), DALYs (AAPC: 1.96%, 95% CI: 1.93% to 1.99%), and a slight rise in mortality (AAPC: 0.41%, 95% CI: 0.37% to 0.44%) (Fig. 1 and Tables S1 and S2).

### Age-specific trends

In 2021, the highest incidence rate in China was observed in the 20 to 24 years age group (522.03, 95% UI: 421.66 to 628.58), with the lowest in the 30 to 34 years age group (170.72, 95% UI: 97.56 to 250.95). DALYs and mortality rates in China also showed an age-dependent increase, with the 35 to 39 years age group bearing the heaviest burden. From 1990 to 2021, both incidence and DALYs for early-onset T2D increased across all age groups in China, with most increases surpassing global rates. The highest AAPC for incidence was in the 15 to 19 years group (4.08%, 95% CI: 3.93% to 4.29%, *P* < 0.001), and for DALYs, it was in the 20 to 24 years group (3.53%, 95% CI: 3.39% to 3.68%, *P* < 0.001). Mortality trends in China were mixed, with a marked decline in the 15 to 19 years group (AAPC: −1.57%, 95% CI: −1.63% to −1.51%, *P* < 0.001) and a slight increase in the 25 to 29 years group (AAPC: 0.39%, 95% CI: 0.30% to 0.50%, *P* < 0.001). In comparison, global trends in 2021 showed the highest incidence rate in the 35 to 39 years age group and a consistent increase across all age groups, with the most pronounced rise in the 15 to 19 years cohort (AAPC: 0.67%, 95% CI: 0.64% to 0.71%, *P* < 0.001). Global DALYs also followed an age-dependent increase, similar to China, but with a generally lower burden (Fig. 1 and Tables S1 and S2).

### Sex-specific trends

**Fig. 2.**
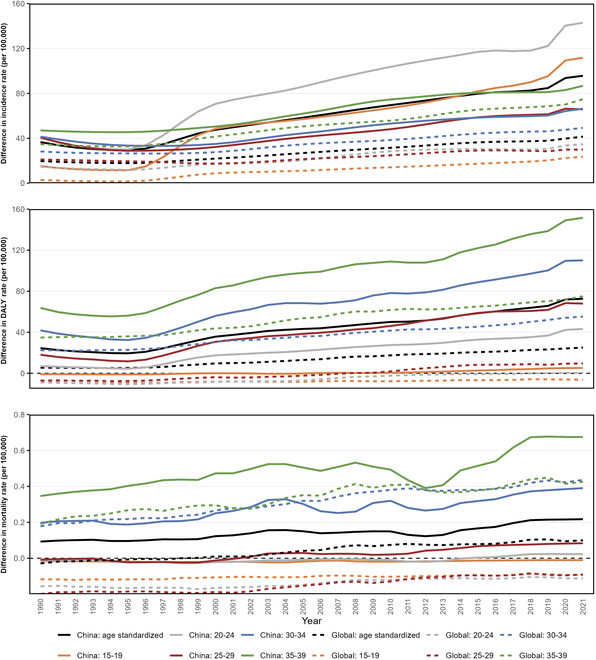
Sex differences in the disease burden of early-onset T2D in China and globally by age groups from 1990 to 2021. The difference is calculated as follows: the age-specific rate in males minus that in females, with a difference >0 meaning males have higher rates.

In 2021, sex-specific trends in the burden of early-onset T2D in China revealed substantial disparities, with males consistently showing higher ASR across all metrics compared to females: incidence [361.21 (95% UI: 261.10 to 476.43) versus 265.51 (95% UI: 187.15 to 355.80) per 100,000], DALYs [302.35 (95% UI: 195.79 to 433.57) versus 229.74 (95% UI: 144.78 to 336.95) per 100,000], and mortality [0.38 (95% UI: 0.29 to 0.49) versus 0.16 (95% UI: 0.12 to 0.22) per 100,000] (Fig. 1 and Tables S1 and S2). From 1990 to 2021, secular trend analysis demonstrated that the burden grew faster in males, with an AAPC for incidence of 2.70% (95% CI: 2.62% to 2.81%, *P* < 0.001) compared to 2.59% (95% CI: 2.51% to 2.66%, *P* < 0.001) in females. For ASR of DALYs, the AAPC was 2.83% (95% CI: 2.72% to 2.95%, *P* < 0.001) in males and 2.63% (95% CI: 2.52% to 2.74%, *P* < 0.001) in females. The sex difference in age-standardized incidence rates expanded from 36.62 to 95.70 per 100,000, while the difference in DALY rates increased from 24.53 to 72.60 per 100,000. Notably, ASR of mortality exhibited opposite trends between sex, with an increase in males (AAPC: 0.43%, 95% CI: 0.31% to 0.56%, *P* < 0.001) and a decrease in females (AAPC: −1.36%, 95% CI: −1.47% to −1.24%, *P* < 0.001), with declines observed across all age groups (Fig. 2).

In comparison, global trends also reflected similar sex differences, though to a lesser extent. The AAPC for global incidence was 2.35% (95% CI: 2.33% to 2.37%, *P* < 0.001) in males and 2.33% (95% CI: 2.30% to 2.36%, *P* < 0.001) in females. Similar patterns were observed for DALYs and mortality, with males consistently showing higher growth rates (Fig. 1 and Tables S1 and S2).

### Secular trend of the proportion of the disease burden from early-onset T2D out of the total burden of T2D

**Fig. 3.**
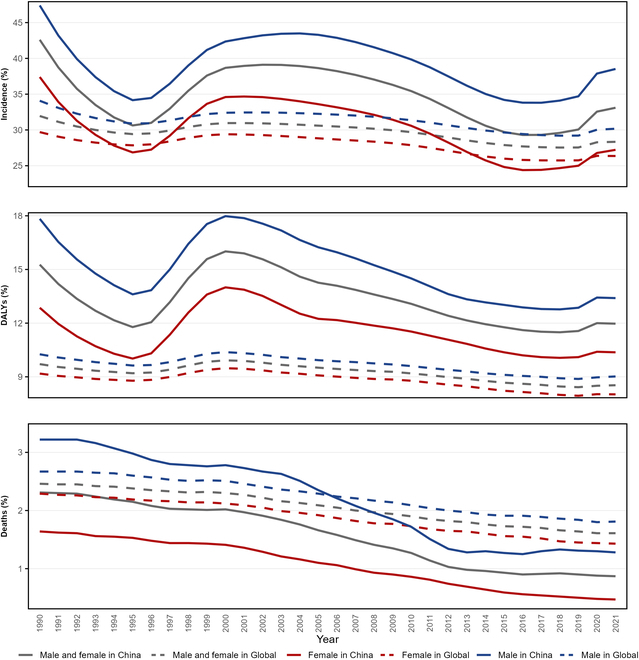
Sex-specific secular trends in proportions of early-onset T2D disease burden within total T2D burden in China and globally, 1990–2021.

From 1990 to 2021, the proportions of early-onset T2D incidence and DALYs within total T2D in China fluctuated but consistently remained higher than global levels. Over these 31 years, the proportion of incidence decreased from 42.60% to 33.10% in China, while the global proportion slightly decreased from 31.95% to 28.34%. The proportion of DALYs also showed a declining trend, dropping from 15.27% to 11.97%, but still remained higher than the global level of 8.53% in 2021. Regarding mortality, both China and global proportions showed downward trends, with China decreasing from 2.31% to 0.87%, a larger decline than the global level (from 2.46% to 1.61%). Notably, since 2006, China's early-onset T2D mortality proportion has been lower than the global level (Fig. 3 and Table S3).

A stratified analysis by sex revealed that the burden of early-onset T2D among Chinese males was consistently higher than among females and exhibited more pronounced fluctuations, while the global level showed relatively smaller sex differences. In 2021, the proportion of early-onset T2D incidence among Chinese males was 38.52%, compared to 30.17% globally, and the proportion of DALYs was 13.40%, compared to 9.02% globally. In contrast, the proportions for Chinese females were closer to the global levels. In 2021, the proportion of early-onset T2D incidence among Chinese females was 27.22%, compared to 26.36% globally, and the proportion of DALYs was 10.37%, compared to 8.02% globally (Fig. 3 and Table S3).

### Proportion of attributable risk factors of DALYs for early-onset T2D and T2D in China in 2021

In 2021, high BMI was the leading contributor to DALYs for early-onset T2D and T2D in the Chinese population, accounting for 59.85% (95% UI: 33.54 to 76.65) and 51.40% (95% UI: 24.11 to 72.31). Other contributors included ambient particulate matter pollution, diet high in red meat, smoking, and diet low in whole grains, which accounted for 14.77% (95% UI: 8.24 to 21.24) versus 16.67% (95% UI: 9.01 to 23.93), 9.33% (95% UI: −1.42 to 20.06) versus 9.28% (95% UI: −1.42 to 20.18), 8.94% (95% UI: 7.59 to 10.30) versus 11.20% (95% UI: 9.44 to 12.83), and 7.89% (95% UI: 2.40 to 13.18) versus 7.43% (95% UI: 2.22 to 12.20) for early-onset T2D and T2D, respectively.

**Fig. 4.**
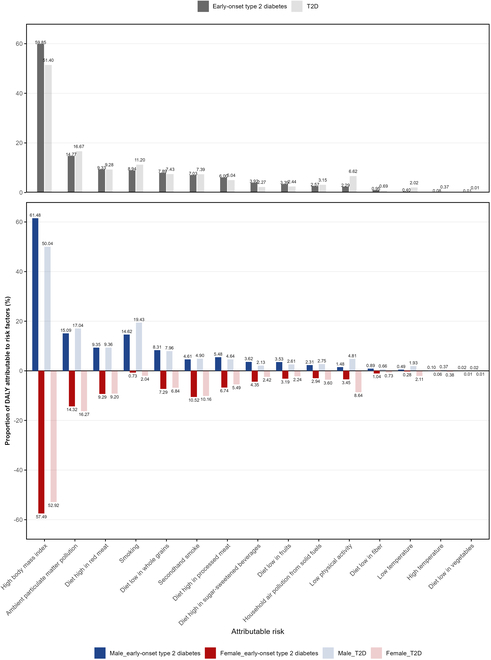
Proportions of attributable risk factors for early-onset T2D and T2D DALYs in China, 2021.

When comparing across sex, high BMI, ambient particulate matter pollution, smoking, diet high in red meat, and diet low in whole grains were the top 5 attributable risk factors for DALYs of both early-onset T2D and T2D in males. For females, the top 5 attributable risk factors for DALYs of early-onset T2D were high BMI, ambient particulate matter pollution, secondhand smoke, diet high in red meat, and diet low in whole grains. For DALYs of T2D in females, the top 4 risk factors were the same as those for early-onset T2D, with low physical activity ranking fifth. Notably, secondhand smoke contributed more substantially to DALYs of early-onset T2D in females (10.52%, 95% UI: 3.88 to 16.68) compared to males (4.61%, 95% UI: 1.62 to 7.90). Conversely, smoking had a greater impact on males with early-onset T2D (14.62%, 95% UI: 12.48 to 16.80) than on females (0.73%, 95% UI: 0.44 to 1.14) (Fig. 4 and Tables S4 and S5).

### Secular trend for proportion of top 5 attributable risk factors of DALYs for early-onset T2D in China from 1990 to 2021

**Fig. 5.**
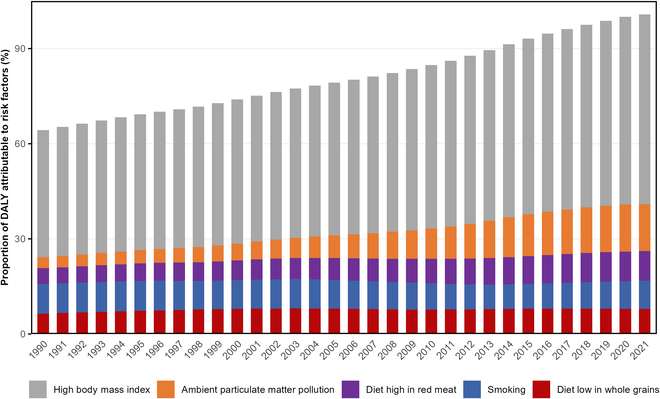
Temporal trends for proportions of the top 5 attributable risk factors of DALYs for early-onset T2D in China, 1990–2021.

The secular trend analysis of the proportional contributions of the top 5 attributable risk factors for DALYs of early-onset T2D in China from 1990 to 2021 reveals marked shifts in the influence of various factors. High BMI consistently emerged as the most important risk factor throughout the period. Its contribution increased substantially from 40.08% (95% UI: 20.71 to 55.79) in 1990 to 59.85% (95% UI: 33.54 to 76.65) in 2021. Ambient particulate matter pollution exhibited a notable upward trend in its contribution to DALYs, rising from 3.46% (95% UI: 1.53 to 6.28) in 1990 to 14.77% (95% UI: 8.24 to 21.24) in 2021, making it the second most important risk factor by 2021. The contribution of a diet high in red meat increased moderately from 4.94% (95% UI: −0.71 to 11.65) in 1990 to 9.33% (95% UI: −1.42 to 20.06) in 2021. Smoking's impact on DALYs remained relatively stable, with a slight decrease from 9.38% (95% UI: 7.88 to 10.92) in 1990 to 8.94% (95% UI: 7.59 to 10.30) in 2021. Finally, the contribution of a diet low in whole grains showed a slight increase from 6.42% (95% UI: 1.79 to 10.55) in 1990 to 7.89% (95% UI: 2.40 to 13.18) in 2021 (Fig. 5 and Table S7).

## Discussion

This study, based on data from the GBD 2021, conducted a comprehensive analysis of the burden of early-onset T2D in China and compared it with global burden data. It revealed marked trends, population distribution characteristics, and attributable risk factors from 1990 to 2021. Our findings highlighted the unique challenges and opportunities that China faces in addressing early-onset T2D. The key findings of our study are summarized as follows. First, during 1990–2021, the ASR of incidence and DALYs in China showed substantial increasing trends, with both rates being higher than the global average. Meanwhile, the ASR of mortality in China slightly decreased, contrasting with the global increasing trend. Second, compared to females, males in China had higher ASR of incidence, DALYs, and mortality rates of early-onset T2D, as well as faster growth. These sex differences were more pronounced in the age groups of 30 to 34 and 35 to 39 years. Third, the DALYs and mortality rates of early-onset T2D in the Chinese population generally increased with age; the 15 to 19 years age group showed the fastest increase in incidence. Fourth, the proportion of the disease burden from early-onset T2D out of the total burden of T2D in China overall showed decreasing trends, while proportions of early-onset T2D annual incident cases and DALYs for T2D were higher in China compared with the global levels, and these higher levels were mainly driven by males in China. Finally, the top 5 attributable risk factors for DALYs of early-onset T2D and T2D in China were almost identical, high BMI made the largest proportional contribution to DALYs of early-onset T2D in China, and this contribution continued to increase during the study period.

This study found that from 1990 to 2021, the ASR of incidence and DALYs of early-onset T2D in China showed substantial increasing trends, with both incidence rate and DALYs being higher than the global average, consistent with previous research findings [8]. When the global data were stratified by sociodemographic index (SDI), China's ASRs for incidence and DALYs were found to be higher than those in countries with a low-middle SDI, where the incidence and DALYs were the highest among all SDI categories. Meanwhile, China's mortality rates were closer to those of high SDI countries, which exhibited the lowest mortality rates across all SDI categories [8]. The relatively high incidence may be related to the rapidly improving economic level and changing lifestyle in China in recent years. Studies have shown that between 1980 and 2010, the dietary pattern was transforming from high-carb, high-fiber, and low-fat diet to a high-fat, high-energy, and low-carb diet in China [29]. Meanwhile, obesity and overweight rates rose rapidly among Chinese adolescents and young adults, while physical activity levels gradually declined [30–32]. These factors collectively form the socioeconomic and behavioral foundation for the rapid growth of early-onset T2D. Regarding mortality, this study found that the age-standardized mortality rate of early-onset T2D in China slightly decreased, whereas globally, it has shown an increasing trend, which is consistent with previous research findings [8]. The relatively lower mortality may be associated with sustained investment in diabetes care in China. Since 2009, the Chinese government has incorporated T2D as one of the 2 major noncommunicable diseases into basic public health services, gradually implementing nationwide screening, regular follow-ups, health education, and other measures [33,34]. One survey showed that from 2009 to 2018, the health management and health education rates for diabetes in China showed increasing trends, and blood glucose control among patients also improved [35]. However, the continued rapid growth in incidence rates and DALYs highlights that major challenges remain in the prevention and control of early-onset T2D. While progress has been made in reducing mortality, there is a pressing need to strengthen primary prevention efforts, particularly among healthy young populations.

The trends of increasing DALYs and mortality with age were consistent in both China and globally. As a progressive disease, the role of age in promoting diabetes incidence has been well recognized [36,37]. This reflects the cumulative effects of disease progression and underscores the importance of early intervention. Although the immediate disease burden may be lighter in younger populations, the long-term health impacts can be more severe if not adequately managed. Additionally, our study found that the AAPC of ASR of incidence was high in patients aged 15 to 19 and 20 to 24 years, both in China and globally, suggesting that prevention and control efforts need to be intensified in these age groups.

Globally, the burden of early-onset T2D exhibits marked sex differences. Xie et al. [8] found that the rate of increase in the diabetes burden is notably higher among males than females worldwide, consistent with our findings in China. This disparity may be attributed to various behavioral, lifestyle, and biological factors. Previous studies have pointed out that compared to females, males pay less attention to dietary nutrition, lack motivation to participate in health promotion activities, are less likely to develop healthy habits, and are less inclined to seek medical services [38,39]. In addition, males also have higher smoking rates [40]. These patterns not only increase the risk of diabetes in males but may also lead to a higher likelihood of diabetes-related complications. Furthermore, biological mechanisms may exacerbate these sex differences. Research suggests that estrogen in females provides protective effects through various mechanisms, including enhancing insulin sensitivity, promoting insulin secretion, and reducing β cell apoptosis, thereby potentially delaying or reducing the onset of T2D [41]. In contrast, males are more prone to visceral fat accumulation, a fat type strongly associated with insulin resistance, which, in turn, increases the risk of developing diabetes [42]. In terms of mortality, this study revealed differences between trends in China and globally. While global data indicate that mortality rates from early-onset T2D are rising among both males and females, with a particularly pronounced increase among males [8], the age-standardized mortality rate in Chinese females, as well as mortality rates across all age groups, has shown a declining trend. This may be partly due to higher diabetes incidence and prevalence in males, and also possibly related to the higher awareness rate, treatment rate, and control rate of diabetes in females in China [43,44]. Moreover, the more active participation of Chinese females in health behaviors, such as more frequent medical checkups and health education, may further contribute to the declining mortality rates in this population [38,39]. The observed differences between China and global trends could be attributed to disparities in healthcare accessibility and the quality of health services across countries. Studies have shown that among diabetic patients under the age of 30, females often have higher mortality rates than males, particularly in countries with a low SDI [8], which may be due to the fact that females in some less-developed regions face greater difficulties in accessing healthcare services and tend to have poorer metabolic health, including higher obesity rates and suboptimal glycemic control [45]. These findings underscore the critical need for targeted, sex-specific interventions in the prevention and management of early-onset T2D in China. For males, it is imperative to enhance education on diabetes prevention and management, increase awareness and treatment adherence, and promote healthier lifestyle modifications.

Regarding the proportion of disease burden from early-onset T2D out of total T2D, previous studies have only reported the proportion of prevalent early-onset T2D cases. For example, results from the Joint Asia Diabetes Evaluation (JADE) cohort showed that the proportion of early-onset T2D prevalent cases among all T2D prevalence cases in China was around 20% [13]. Our study analyzed the proportions of early-onset T2D annual incidence cases, DALYs, and deaths, and the results showed that these proportions decreased from 1990 to 2021. This may be related to the following 2 aspects. On the one hand, the aging of the Chinese population may have led to a more rapid increase in the disease burden of T2D among the middle-aged and elderly population. A previous study showed that in China, T2D deaths attributed to population aging and population growth increased gradually from 1991 to 2019, with 1990 as the baseline[44]. On the other hand, it is possible that the current Chinese guidelines for the prevention and treatment of T2D, which recommend diabetes screening primarily for people aged 40 and above [46], may lead to underdiagnosis in younger individuals with T2D. In addition, it is noteworthy that although the proportion of disease burden from early-onset T2D out of total T2D in China showed a decreasing trend, the current value of this proportion in China was still higher than the global level, especially among males. This means that the prevention and control of early-onset T2D remains important in China, with males being a key target population.

Consistent with global results reported by a previous study, our study found that high BMI was the main contributing risk factor for early-onset T2D, and its proportional contribution continued to increase over time [8]. This phenomenon has also been observed in the study of the disease burden of diabetes across all age groups in China [47]. In recent years, with the economic development, the problem of overweight and obesity among children and adolescents has become increasingly serious in China. Studies showed that between 1991 and 2015, an overall upward trend was observed in the rates of overweight and obesity among Chinese children and adolescents [48]. This suggested that controlling body weight among children and adolescents may be important to preventing further increases in the disease burden of early-onset T2D in China. In addition, our study also reported the increasing contribution of ambient particulate matter pollution to early-onset T2D. Previous studies have reported that after extensive efforts to control emissions, pollution from ambient particulate matter decreased overall in recent years in China. However, because of rapid urbanization in China, the growing migration of people into more polluted cities has led to the continued rise in the contribution of ambient particulate matter pollution [49,50]. This underscores the importance of paying special attention to environmental factors when addressing early-onset T2D in China.

To our knowledge, our study is the first to analyze the secular trend in the disease burden of early-onset T2D in China. We provided a comprehensive description of the population distribution characteristics and attributable risk factors in addition to analyzing the secular trend using data from the GBD 2021. However, our study also has some limitations. First, our study was a secondary analysis based on GBD 2021. Although the GBD study provides widely used standardized methods and data for global health assessments, there are still limitations in data quality for nonfatal health outcomes in most countries. Thus, unavoidable biases may exist due to limitations of the GBD data, such as the potential bias in defining T2D by subtracting cases definitively reported as type 1 diabetes from total diabetes cases. Therefore, our findings should be interpreted with caution, and future real-world studies in China are needed to validate our results. Second, due to the lack of diabetes data for populations under 15 years old in the GBD data, we could only define the early-onset T2D as patients with T2D aged 15 to 39 years, which differs somewhat from the general definition of early-onset T2D. However, since the incidence of T2D in populations below 15 years old is low, we believe that the lack of this population may not greatly affect the trends, distribution characteristics, and attributable risk factors of the disease burden of early-onset T2D in China in our results [51]. Additionally, the GBD 2021 dataset does not support the detailed analysis of specific complications attributable to T2D, such as cardiovascular diseases or blindness, limiting our ability to explore these aspects. Finally, as the GBD 2021 did not directly provide provincial data in China, we were unable to describe the geographical distribution of early-onset T2D across different regions in China, which needs further studies in the future.

## Conclusion

The disease burden of early-onset T2D in China showed an overall upward trend, with the incidence rate already higher than the global incidence, indicating a need to strengthen primary prevention of early-onset T2D among healthy populations. Males and populations over 30 years old had relatively higher disease burdens, with the highest incidence rate observed in the 20 to 24 years age group and the fastest increase in the 15 to 19 years age group, suggesting that timely preventative measures should target these populations, with a particular focus on sex-specific interventions. Additionally, high BMI was the main attributable risk factor for early-onset T2D in China; thus, controlling overweight and obesity will be the key for China to subsequently control and prevent early-onset T2D going forward.

## Data Availability

The data that supported the findings of this study can be obtained from the Global Health Data Exchange Global Burden of Disease Results Tool (https://ghdx.healthdata.org/gbd-results-tool).
