# Supplementary material for: The Burden of Type 2 Diabetes in Adolescents and Young Adults in China: A Secondary Analysis from the Global Burden of Disease Study 2021
Source: Health Data Sci. 2024 Dec 17;4:0210. doi: 10.34133/hds.0210 (PMC11651706; doi:10.34133/hds.0210)
Supplement: Supplementary 1 — Tables S1 to S8 [file hds.0210.f1.docx]

**Appendix**

[**Appendix** 1](#_Toc173963850)

[Supplementary Table 1 Disease burden for early-onset type 2 diabetes by age groups and sexes in 1990 and 2021 in China 2](#_Toc173963851)

[Supplementary Table 2 Disease burden for early-onset type 2 diabetes by age groups and sexes in 1990 and 2021 in Global 4](#_Toc173963852)

[Supplementary Table 3 Proportion of the disease burden from early-onset type 2 diabetes out of the total burden of T2D in China and Global during 1990 to 2021 6](#_Toc173963853)

[Supplementary Table 4 Proportion of attributable risk factors for DALY burden of early-onset type 2 diabetes and all type 2 diabetes in China and Global, 2021 9](#_Toc173963854)

[Supplementary Table 5 Proportion of attributable risk factors for DALY burden of early-onset type 2 diabetes and T2D in China by sex, 2021 10](#_Toc173963855)

[Supplementary Table 6 Proportion of attributable risk factors for DALY burden of early-onset type 2 diabetes and T2D in Global by sex,2021 11](#_Toc173963856)

[Supplementary Table 7 Temporal trends for the proportion of the top five attributable risk factors of DALYs for early-onset type 2 diabetes in China from 1990 to 2021 12](#_Toc173963857)

[Supplementary Table 8 Temporal trends for the proportion of the top five attributable risk factors of DALYs for early-onset type 2 diabetes in Global from 1990 to 2021 14](#_Toc173963858)

# Supplementary Table 1 Disease burden for early-onset type 2 diabetes by age groups and sexes in 1990 and 2021 in China

| **Age groups** | **Both sexes** | | | | **Men** | | | | **Women** | | | |
| --- | --- | --- | --- | --- | --- | --- | --- | --- | --- | --- | --- | --- |
|  | **Rates in 1990**  **(95% UI)** | **Rate in 2021**  **(95% UI)** | **AAPC, %**  **(95% CI)** | ***P*** | **Rates in 1990**  **(95% UI)** | **Rate in 2021**  **(95% UI)** | **AAPC, %**  **(95% CI)** | ***P*** | **Rates in 1990**  **(95% UI)** | **Rate in 2021**  **(95% UI)** | **AAPC, %**  **(95% CI)** | ***P*** |
| Incidence | | | | | | | | | | | | |
| Age-standardized | 140.20 (89.14, 204.74) | 315.97 (226.75, 417.55) | 2.67 (2.60, 2.75) | <0.001 | 157.94 (100.49, 230.51) | 361.21 (261.1, 476.43) | 2.70 (2.62, 2.81) | <0.001 | 121.32 (76.75, 177.57) | 265.51 (187.15, 355.8) | 2.59 (2.51, 2.66) | <0.001 |
| 15-19 years | 128.66 (85.67, 175.62) | 439.85 (336.24, 552.63) | 4.08 (3.93, 4.29) | <0.001 | 136.07 (89.77, 187.42) | 491.6 (378.73, 621.45) | 4.37 (4.23, 4.53) | <0.001 | 120.83 (81.98, 163.69) | 379.89 (285.36, 477.82) | 3.81 (3.66, 4) | <0.001 |
| 20-24 years | 164.43 (116.51, 225.89) | 522.03 (421.66, 628.58) | 3.92 (3.77, 4.08) | <0.001 | 184.64 (131.66, 252.9) | 589.05 (480.9, 705.2) | 3.85 (3.7, 4.03) | <0.001 | 143.27 (99.69, 196.36) | 446.09 (350.32, 543.74) | 3.89 (3.72, 4.05) | <0.001 |
| 25-29 years | 124.71 (73.51, 186.73) | 212.47 (124.62, 318.72) | 1.75 (1.69, 1.82) | <0.001 | 144.44 (84.47, 214.1) | 243.58 (143.67, 363.04) | 1.71 (1.65, 1.78) | <0.001 | 103.89 (61.39, 156.91) | 177.74 (102.77, 274.08) | 1.79 (1.72, 1.85) | <0.001 |
| 30-34 years | 126.34 (74.51, 191.75) | 170.72 (97.56, 250.95) | 0.99 (0.95, 1.03) | <0.001 | 146.17 (86.88, 221.73) | 202.74 (119.06, 295.17) | 1.08 (1.03, 1.12) | <0.001 | 104.72 (60.95, 159.8) | 136.39 (77.01, 207.9) | 0.88 (0.84, 0.92) | <0.001 |
| 35-39 years | 157.95 (94.72, 248.74) | 201.69 (123.75, 301.87) | 0.80 (0.78, 0.83) | <0.001 | 180.65 (109.62, 283.35) | 243.94 (150.5, 360.31) | 0.98 (0.96, 1) | <0.001 | 133.65 (78.03, 214.25) | 157.17 (94.07, 243.24) | 0.54 (0.5, 0.58) | <0.001 |
| DALY | | | | | | | | | | | | |
| Age-standardized | 116.29 (78.51, 167.05) | 267.47 (171.08, 387.38) | 2.75 (2.64, 2.87) | <0.001 | 128.12 (86.45, 184.29) | 302.35 (195.79, 433.57) | 2.83 (2.72, 2.95) | <0.001 | 103.59 (69.9, 149.37) | 229.74 (144.78, 336.95) | 2.63 (2.52, 2.74) | <0.001 |
| 15-19 years | 28.15 (17.7, 44.61) | 54.19 (33.08, 84.03) | 2.15 (2.05, 2.25) | <0.001 | 27.69 (17.12, 44.15) | 56.6 (34.3, 87.33) | 2.33 (2.22, 2.45) | <0.001 | 28.63 (17.83, 45.77) | 51.4 (31.74, 79.94) | 1.90 (1.8, 2.01) | <0.001 |
| 20-24 years | 72.51 (46.45, 106.54) | 205.36 (124.26, 305.51) | 3.53 (3.39, 3.68) | <0.001 | 75.94 (48.64, 112.51) | 225.63 (137.53, 334.18) | 3.71 (3.57, 3.86) | <0.001 | 68.93 (44.57, 101.71) | 182.39 (111.09, 277.69) | 3.24 (3.1, 3.38) | <0.001 |
| 25-29 years | 111.19 (71.28, 163.43) | 297.25 (184.35, 430.81) | 3.27 (3.15, 3.39) | <0.001 | 119.97 (74.6, 176.53) | 329.35 (203.48, 472.05) | 3.41 (3.24, 3.59) | <0.001 | 101.93 (66.41, 147.62) | 261.42 (160.29, 380.94) | 3.13 (3.03, 3.25) | <0.001 |
| 30-34 years | 162.35 (111.99, 233.15) | 367.76 (237.34, 531.75) | 2.69 (2.57, 2.82) | <0.001 | 182.35 (126.22, 260.4) | 420.87 (277.4, 605.74) | 2.76 (2.66, 2.89) | <0.001 | 140.55 (95.49, 203.04) | 310.82 (195.87, 453.56) | 2.64 (2.55, 2.72) | <0.001 |
| 35-39 years | 227.67 (159.81, 315.29) | 451.76 (303.14, 639.03) | 2.31 (2.22, 2.39) | <0.001 | 258.41 (182.87, 360.42) | 525.55 (358.65, 732.07) | 2.39 (2.3, 2.48) | <0.001 | 194.76 (137.33, 271.67) | 374.01 (245.84, 536.62) | 2.19 (2.11, 2.27) | <0.001 |
| Mortality | | | | | | | | | | | | |
| Age-standardized | 0.30 (0.24, 0.38) | 0.28 (0.23, 0.34) | -0.22 (-0.33, -0.11) | <0.001 | 0.35 (0.26, 0.45) | 0.38 (0.29, 0.49) | 0.43 (0.31, 0.56) | <0.001 | 0.25 (0.17, 0.35) | 0.16 (0.12, 0.22) | -1.36 (-1.47, -1.24) | <0.001 |
| 15-19 years | 0.06 (0.04, 0.08) | 0.04 (0.03, 0.05) | -1.57 (-1.63, -1.51) | <0.001 | 0.05 (0.04, 0.07) | 0.03 (0.02, 0.05) | -1.61 (-1.68, -1.56) | <0.001 | 0.07 (0.05, 0.1) | 0.04 (0.03, 0.06) | -1.53 (-1.59, -1.46) | <0.001 |
| 20-24 years | 0.12 (0.09, 0.17) | 0.11 (0.09, 0.14) | -0.17 (-0.32, -0.03) | 0.02 | 0.12 (0.08, 0.17) | 0.12 (0.09, 0.17) | 0.24 (0.06, 0.41) | 0.01 | 0.13 (0.08, 0.19) | 0.10 (0.08, 0.14) | -0.83 (-0.92, -0.73) | <0.001 |
| 25-29 years | 0.15 (0.11, 0.2) | 0.16 (0.13, 0.21) | 0.39 (0.3, 0.5) | <0.001 | 0.15 (0.1, 0.2) | 0.20 (0.16, 0.26) | 1.11 (1, 1.21) | <0.001 | 0.15 (0.1, 0.22) | 0.12 (0.09, 0.17) | -0.63 (-0.79, -0.48) | <0.001 |
| 30-34 years | 0.43 (0.34, 0.55) | 0.41 (0.34, 0.51) | -0.10 (-0.2, 0.01) | 0.07 | 0.52 (0.4, 0.67) | 0.60 (0.47, 0.75) | 0.47 (0.35, 0.59) | <0.001 | 0.33 (0.22, 0.48) | 0.21 (0.15, 0.29) | -1.37 (-1.5, -1.25) | <0.001 |
| 35-39 years | 0.82 (0.67, 1.01) | 0.73 (0.6, 0.89) | -0.40 (-0.52, -0.31) | <0.001 | 0.99 (0.76, 1.24) | 1.06 (0.81, 1.35) | 0.22 (0.13, 0.29) | <0.001 | 0.64 (0.45, 0.86) | 0.38 (0.29, 0.51) | -1.62 (-1.68, -1.53) | <0.001 |

UI: Uncertainty Interval, AAPC: Average Annual Percent Change.

# Supplementary Table 2 Disease burden for early-onset type 2 diabetes by age groups and sexes in 1990 and 2021 in Global

| **Age groups** | **Both sexes** | | | | **Men** | | | | **Women** | | | |
| --- | --- | --- | --- | --- | --- | --- | --- | --- | --- | --- | --- | --- |
|  | **Rates in 1990**  **(95% UI)** | **Rate in 2021**  **(95% UI)** | **AAPC, %**  **(95% CI)** | ***P*** | **Rates in 1990**  **(95% UI)** | **Rate in 2021**  **(95% UI)** | **AAPC, %**  **(95% CI)** | ***P*** | **Rates in 1990**  **(95% UI)** | **Rate in 2021**  **(95% UI)** | **AAPC, %**  **(95% CI)** | ***P*** |
| Incidence | | | | | | | | | | | | |
| Age-standardized | 111.17 (76.3, 152.15) | 226.52 (163.95, 295.73) | 2.34 (2.32, 2.37) | <0.001 | 120.90 (82.98, 164.95) | 246.98 (179.66, 321.00) | 2.35 (2.33, 2.37) | <0.001 | 101.21 (69.24, 139.38) | 205.52 (147.99, 270.10) | 2.33 (2.30, 2.36) | <0.001 |
| 15-19 years | 72.90 (47.3, 99.38) | 167.10 (123.39, 214.37) | 2.73 (2.67, 2.79) | <0.001 | 74.32 (47.77, 101.70) | 178.55 (133.03, 229.72) | 2.89 (2.82, 2.95) | <0.001 | 71.43 (47.03, 97.2) | 155.03 (113.74, 199.01) | 2.54 (2.49, 2.59) | <0.001 |
| 20-24 years | 99.19 (70.02, 136.79) | 216.93 (162.92, 278.67) | 2.56 (2.52, 2.62) | <0.001 | 106.44 (74.25, 146.89) | 233.98 (176.60, 298.11) | 2.58 (2.53, 2.64) | <0.001 | 91.82 (64.65, 126.6) | 199.31 (148.33, 259.36) | 2.53 (2.49, 2.58) | <0.001 |
| 25-29 years | 101.07 (66.74, 140.62) | 200.69 (135.97, 271.69) | 2.25 (2.24, 2.27) | <0.001 | 111.59 (73.86, 154.76) | 215.53 (145.54, 292.94) | 2.16 (2.14, 2.18) | <0.001 | 90.45 (59.7, 126.59) | 185.54 (125.6, 251.64) | 2.36 (2.33, 2.38) | <0.001 |
| 30-34 years | 126.24 (87.85, 166.57) | 242.39 (174.84, 312.22) | 2.14 (2.12, 2.16) | <0.001 | 140.16 (98.27, 184.51) | 266.77 (193.07, 341.35) | 2.11 (2.09, 2.12) | <0.001 | 111.94 (77.11, 148.95) | 217.46 (156.20, 282.93) | 2.18 (2.14, 2.20) | <0.001 |
| 35-39 years | 165.43 (116.18, 229.76) | 319.71 (232.64, 420.84) | 2.17 (2.14, 2.19) | <0.001 | 182.52 (128.58, 251.14) | 356.78 (261.99, 464.92) | 2.20 (2.18, 2.21) | <0.001 | 147.81 (103.03, 208.02) | 281.94 (204.29, 373.46) | 2.12 (2.09, 2.15) | <0.001 |
| DALY | | | | | | | | | | | | |
| Age-standardized | 116.50 (89.38, 150.34) | 211.92 (153.93, 285.16) | 1.96 (1.93, 1.99) | <0.001 | 119.16 (90.21, 155.34) | 224.32 (162.56, 303.94) | 2.06 (2.03, 2.09) | <0.001 | 113.73 (88.19, 146.69) | 199.29 (145.12, 266.72) | 1.84 (1.08, 1.87) | <0.001 |
| 15-19 years | 36.94 (28.95, 48.52) | 60.53 (46.28, 81.52) | 1.61 (1.58, 1.63) | <0.001 | 32.52 (25.14, 44.05) | 57.53 (43.26, 79.66) | 1.86 (1.84, 1.88) | <0.001 | 41.51 (32.44, 54.7) | 63.69 (47.66, 85.54) | 1.39 (1.37, 1.42) | <0.001 |
| 20-24 years | 63.39 (47.19, 85.28) | 117.15 (82.81, 160.82) | 2.00 (1.97, 2.03) | <0.001 | 58.98 (42.45, 80.78) | 117.41 (82.4, 163.66) | 2.24 (2.22, 2.28) | <0.001 | 67.88 (50.35, 89.36) | 116.87 (83.73, 158.12) | 1.77 (1.74, 1.81) | <0.001 |
| 25-29 years | 96.60 (70.77, 127.75) | 186.01 (129.35, 253.16) | 2.15 (2.12, 2.18) | <0.001 | 93.06 (65.04, 126.45) | 190.76 (131.90, 262.64) | 2.34 (2.31, 2.37) | <0.001 | 100.19 (76.00, 131.51) | 181.15 (127.88, 243.11) | 1.93 (1.89, 1.96) | <0.001 |
| 30-34 years | 161.58 (125.32, 206.35) | 297.48 (215.24, 400.99) | 2.00 (1.95, 2.03) | <0.001 | 172.65 (133.51, 220.88) | 324.82 (237.16, 438.67) | 2.08 (2.04, 2.11) | <0.001 | 150.22 (118.19, 192.43) | 269.54 (194.82, 362.82) | 1.92 (1.88, 1.96) | <0.001 |
| 35-39 years | 245.87 (191.84, 311.19) | 437.89 (325.2, 581.54) | 1.90 (1.87, 1.92) | <0.001 | 263.02 (204.02, 335.2) | 475.06 (350.67, 633.28) | 1.94 (1.91, 1.97) | <0.001 | 228.18 (179.32, 289.74) | 400.01 (297.36, 530.1) | 1.85 (1.82, 1.88) | <0.001 |
| Mortality | | | | | | | | | | | | |
| Age-standardized | 0.75 (0.68, 0.82) | 0.85 (0.76, 0.96) | 0.41 (0.37, 0.44) | <0.001 | 0.74 (0.65, 0.82) | 0.90 (0.78, 1.05) | 0.64 (0.59, 0.69) | <0.001 | 0.77 (0.67, 0.86) | 0.80 (0.70, 0.90) | 0.15 (0.12, 0.17) | <0.001 |
| 15-19 years | 0.28 (0.24, 0.31) | 0.34 (0.29, 0.39) | 0.67 (0.64, 0.71) | <0.001 | 0.22 (0.18, 0.25) | 0.30 (0.24, 0.37) | 0.98 (0.94, 1.01) | <0.001 | 0.34 (0.28, 0.39) | 0.39 (0.32, 0.46) | 0.44 (0.38, 0.49) | <0.001 |
| 20-24 years | 0.34 (0.30, 0.38) | 0.40 (0.34, 0.46) | 0.53 (0.49, 0.56) | <0.001 | 0.26 (0.22, 0.31) | 0.35 (0.28, 0.43) | 0.88 (0.84, 0.92) | <0.001 | 0.42 (0.35, 0.48) | 0.46 (0.38, 0.52) | 0.29 (0.24, 0.32) | <0.001 |
| 25-29 years | 0.49 (0.43, 0.54) | 0.56 (0.49, 0.65) | 0.49 (0.44, 0.55) | <0.001 | 0.39 (0.33, 0.44) | 0.51 (0.43, 0.62) | 0.94 (0.87, 1.01) | <0.001 | 0.59 (0.50, 0.67) | 0.60 (0.51, 0.69) | 0.16 (0.09, 0.22) | <0.001 |
| 30-34 years | 1.08 (0.98, 1.16) | 1.18 (1.06, 1.31) | 0.24 (0.15, 0.29) | <0.001 | 1.16 (1.05, 1.27) | 1.40 (1.22, 1.60) | 0.49 (0.43, 0.55) | <0.001 | 0.99 (0.87, 1.09) | 0.96 (0.84, 1.07) | -0.18 (-0.25, -0.12) | <0.001 |
| 35-39 years | 1.74 (1.6, 1.87) | 1.96 (1.78, 2.17) | 0.38 (0.35, 0.41) | <0.001 | 1.84 (1.65, 2.02) | 2.17 (1.92, 2.47) | 0.53 (0.50, 0.56) | <0.001 | 1.65 (1.46, 1.82) | 1.75 (1.56, 1.93) | 0.21 (0.17, 0.25) | <0.001 |

UI: Uncertainty Interval, AAPC: Average Annual Percent Change.

# Supplementary Table 3 Proportion of the disease burden from early-onset type 2 diabetes out of the total burden of T2D in China and Global during 1990 to 2021

| **Year** | **China** | | | **Global** | | |
| --- | --- | --- | --- | --- | --- | --- |
|  | **Both sexes** | **Men** | **Women** | **Both sexes** | **Men** | **Women** |
| **Proportions of early-onset type 2 diabetes annual number of incident cases out of all T2D (%)** | | | | | | |
| 1990 | 42.60 | 47.40 | 37.40 | 31.95 | 34.08 | 29.70 |
| 1991 | 38.74 | 43.20 | 33.92 | 31.13 | 33.08 | 29.06 |
| 1992 | 35.75 | 39.92 | 31.28 | 30.48 | 32.27 | 28.58 |
| 1993 | 33.49 | 37.38 | 29.32 | 30.00 | 31.66 | 28.23 |
| 1994 | 31.75 | 35.42 | 27.81 | 29.64 | 31.19 | 27.98 |
| 1995 | 30.63 | 34.16 | 26.86 | 29.42 | 30.89 | 27.85 |
| 1996 | 30.99 | 34.47 | 27.25 | 29.52 | 30.94 | 27.99 |
| 1997 | 32.97 | 36.46 | 29.19 | 29.92 | 31.33 | 28.4 |
| 1998 | 35.48 | 39.01 | 31.63 | 30.41 | 31.82 | 28.89 |
| 1999 | 37.60 | 41.18 | 33.65 | 30.79 | 32.22 | 29.26 |
| 2000 | 38.68 | 42.36 | 34.6 | 30.96 | 32.40 | 29.40 |
| 2001 | 38.95 | 42.82 | 34.67 | 30.96 | 32.44 | 29.36 |
| 2002 | 39.11 | 43.22 | 34.59 | 30.92 | 32.44 | 29.28 |
| 2003 | 39.09 | 43.44 | 34.34 | 30.84 | 32.41 | 29.15 |
| 2004 | 38.93 | 43.49 | 34.00 | 30.73 | 32.34 | 29.00 |
| 2005 | 38.64 | 43.29 | 33.60 | 30.60 | 32.25 | 28.83 |
| 2006 | 38.22 | 42.87 | 33.16 | 30.48 | 32.14 | 28.67 |
| 2007 | 37.69 | 42.28 | 32.68 | 30.34 | 32.01 | 28.52 |
| 2008 | 37.04 | 41.55 | 32.11 | 30.17 | 31.84 | 28.36 |
| 2009 | 36.30 | 40.74 | 31.43 | 29.96 | 31.63 | 28.15 |
| 2010 | 35.43 | 39.85 | 30.58 | 29.69 | 31.36 | 27.87 |
| 2011 | 34.34 | 38.76 | 29.49 | 29.33 | 31.03 | 27.50 |
| 2012 | 33.06 | 37.48 | 28.23 | 28.94 | 30.65 | 27.09 |
| 2013 | 31.75 | 36.18 | 26.92 | 28.54 | 30.27 | 26.67 |
| 2014 | 30.59 | 35.03 | 25.75 | 28.18 | 29.92 | 26.29 |
| 2015 | 29.71 | 34.20 | 24.82 | 27.89 | 29.65 | 25.99 |
| 2016 | 29.30 | 33.81 | 24.39 | 27.70 | 29.43 | 25.81 |
| 2017 | 29.31 | 33.80 | 24.42 | 27.59 | 29.28 | 25.75 |
| 2018 | 29.59 | 34.10 | 24.67 | 27.54 | 29.20 | 25.74 |
| 2019 | 30.06 | 34.70 | 25.00 | 27.55 | 29.21 | 25.75 |
| 2020 | 32.56 | 37.88 | 26.78 | 28.28 | 30.03 | 26.38 |
| 2021 | 33.10 | 38.52 | 27.22 | 28.34 | 30.17 | 26.36 |
| **Proportions of early-onset type 2 diabetes annual number of DALYs out of all T2D (%)** | | | | | | |
| 1990 | 15.27 | 17.83 | 12.86 | 9.71 | 10.26 | 9.18 |
| 1991 | 14.19 | 16.54 | 11.96 | 9.55 | 10.08 | 9.05 |
| 1992 | 13.36 | 15.56 | 11.26 | 9.45 | 9.95 | 8.97 |
| 1993 | 12.69 | 14.77 | 10.71 | 9.34 | 9.82 | 8.88 |
| 1994 | 12.16 | 14.12 | 10.29 | 9.27 | 9.73 | 8.83 |
| 1995 | 11.78 | 13.61 | 10.02 | 9.20 | 9.63 | 8.78 |
| 1996 | 12.05 | 13.84 | 10.31 | 9.24 | 9.66 | 8.83 |
| 1997 | 13.17 | 15.00 | 11.35 | 9.40 | 9.82 | 8.99 |
| 1998 | 14.51 | 16.43 | 12.59 | 9.63 | 10.06 | 9.21 |
| 1999 | 15.58 | 17.54 | 13.60 | 9.84 | 10.29 | 9.39 |
| 2000 | 16.01 | 17.98 | 14.00 | 9.92 | 10.38 | 9.48 |
| 2001 | 15.90 | 17.87 | 13.87 | 9.89 | 10.32 | 9.45 |
| 2002 | 15.57 | 17.56 | 13.52 | 9.79 | 10.23 | 9.36 |
| 2003 | 15.12 | 17.18 | 13.02 | 9.67 | 10.10 | 9.24 |
| 2004 | 14.60 | 16.65 | 12.53 | 9.59 | 10.02 | 9.17 |
| 2005 | 14.26 | 16.24 | 12.24 | 9.51 | 9.93 | 9.08 |
| 2006 | 14.09 | 15.96 | 12.17 | 9.44 | 9.87 | 9.01 |
| 2007 | 13.86 | 15.62 | 12.02 | 9.38 | 9.82 | 8.94 |
| 2008 | 13.60 | 15.24 | 11.86 | 9.32 | 9.75 | 8.88 |
| 2009 | 13.35 | 14.88 | 11.71 | 9.28 | 9.69 | 8.85 |
| 2010 | 13.08 | 14.50 | 11.53 | 9.19 | 9.60 | 8.78 |
| 2011 | 12.74 | 14.06 | 11.30 | 9.09 | 9.49 | 8.67 |
| 2012 | 12.41 | 13.62 | 11.07 | 8.98 | 9.39 | 8.56 |
| 2013 | 12.15 | 13.33 | 10.84 | 8.89 | 9.30 | 8.47 |
| 2014 | 11.94 | 13.16 | 10.58 | 8.77 | 9.19 | 8.34 |
| 2015 | 11.77 | 13.02 | 10.37 | 8.67 | 9.11 | 8.22 |
| 2016 | 11.61 | 12.88 | 10.20 | 8.61 | 9.05 | 8.15 |
| 2017 | 11.52 | 12.79 | 10.10 | 8.55 | 9.00 | 8.08 |
| 2018 | 11.49 | 12.77 | 10.06 | 8.46 | 8.92 | 7.99 |
| 2019 | 11.56 | 12.86 | 10.10 | 8.42 | 8.88 | 7.94 |
| 2020 | 12.00 | 13.43 | 10.40 | 8.50 | 8.97 | 8.03 |
| 2021 | 11.97 | 13.40 | 10.37 | 8.53 | 9.02 | 8.02 |
| **Proportions of early-onset type 2 diabetes annual number of deaths out of all T2D (%)** | | | | | | |
| 1990 | 2.31 | 3.22 | 1.64 | 2.46 | 2.67 | 2.29 |
| 1991 | 2.30 | 3.22 | 1.62 | 2.45 | 2.67 | 2.27 |
| 1992 | 2.29 | 3.22 | 1.61 | 2.45 | 2.67 | 2.26 |
| 1993 | 2.24 | 3.16 | 1.56 | 2.42 | 2.65 | 2.23 |
| 1994 | 2.19 | 3.07 | 1.55 | 2.41 | 2.64 | 2.22 |
| 1995 | 2.15 | 2.98 | 1.53 | 2.38 | 2.60 | 2.19 |
| 1996 | 2.08 | 2.87 | 1.48 | 2.35 | 2.57 | 2.17 |
| 1997 | 2.03 | 2.80 | 1.44 | 2.33 | 2.53 | 2.16 |
| 1998 | 2.02 | 2.78 | 1.44 | 2.31 | 2.51 | 2.14 |
| 1999 | 2.01 | 2.76 | 1.43 | 2.32 | 2.52 | 2.14 |
| 2000 | 2.02 | 2.78 | 1.41 | 2.30 | 2.51 | 2.12 |
| 2001 | 1.97 | 2.73 | 1.36 | 2.27 | 2.46 | 2.09 |
| 2002 | 1.91 | 2.67 | 1.29 | 2.22 | 2.41 | 2.05 |
| 2003 | 1.84 | 2.63 | 1.21 | 2.16 | 2.36 | 1.99 |
| 2004 | 1.76 | 2.51 | 1.16 | 2.13 | 2.33 | 1.96 |
| 2005 | 1.66 | 2.35 | 1.10 | 2.09 | 2.29 | 1.92 |
| 2006 | 1.58 | 2.21 | 1.06 | 2.05 | 2.24 | 1.87 |
| 2007 | 1.49 | 2.08 | 0.99 | 2.00 | 2.21 | 1.82 |
| 2008 | 1.41 | 1.96 | 0.93 | 1.97 | 2.17 | 1.78 |
| 2009 | 1.35 | 1.85 | 0.90 | 1.94 | 2.14 | 1.77 |
| 2010 | 1.27 | 1.72 | 0.86 | 1.90 | 2.09 | 1.73 |
| 2011 | 1.14 | 1.51 | 0.81 | 1.85 | 2.04 | 1.68 |
| 2012 | 1.03 | 1.34 | 0.74 | 1.82 | 2.00 | 1.65 |
| 2013 | 0.98 | 1.28 | 0.69 | 1.80 | 1.97 | 1.64 |
| 2014 | 0.96 | 1.30 | 0.64 | 1.76 | 1.93 | 1.60 |
| 2015 | 0.93 | 1.27 | 0.59 | 1.73 | 1.91 | 1.56 |
| 2016 | 0.90 | 1.25 | 0.56 | 1.72 | 1.91 | 1.55 |
| 2017 | 0.91 | 1.30 | 0.54 | 1.70 | 1.89 | 1.52 |
| 2018 | 0.92 | 1.33 | 0.52 | 1.66 | 1.86 | 1.47 |
| 2019 | 0.90 | 1.31 | 0.50 | 1.64 | 1.84 | 1.45 |
| 2020 | 0.88 | 1.30 | 0.48 | 1.61 | 1.80 | 1.44 |
| 2021 | 0.87 | 1.28 | 0.47 | 1.61 | 1.81 | 1.43 |

DALYs: Disability-Adjusted Life Years, T2D: Type 2 diabetes.

# Supplementary Table 4 Proportion of attributable risk factors for DALY burden of early-onset type 2 diabetes and all type 2 diabetes in China and Global, 2021

| **Attributable risk factor** | **China ,% (95% UI)** | | **Global ,% (95% UI)** | |
| --- | --- | --- | --- | --- |
|  | **Early-onset type 2 diabetes** | **T2D** | **Early-onset type 2 diabetes** | **T2D** |
| High body-mass index | 59.85(33.54, 76.65) | 51.40(24.11, 72.31) | 57.21(32.47, 72.69) | 52.15(24.82, 71.80) |
| Ambient particulate matter pollution | 14.77(8.24, 21.24) | 16.67(9.01, 23.93) | 10.04(5.81, 14.76) | 11.83(6.77, 17.36) |
| Diet high in red meat | 9.33(-1.42, 20.06) | 9.28(-1.42, 20.18) | 4.56(-0.66, 10.01) | 5.06(-0.74, 11.32) |
| Smoking | 8.94(7.59, 10.30) | 11.20(9.44, 12.83) | 5.66(4.83, 6.53) | 7.25(6.06, 8.54) |
| Diet low in whole grains | 7.89(2.40, 13.18) | 7.43(2.22, 12.20) | 6.72(1.93, 10.84) | 6.73(1.95, 11.02) |
| Secondhand smoke | 7.03(2.54, 11.45) | 7.39(2.76, 11.93) | 4.78(1.77, 7.90) | 4.89(1.77, 8.03) |
| Diet high in processed meat | 6.00(1.45, 10.35) | 5.04(1.22, 8.78) | 7.03(1.69, 11.62) | 8.08(1.96, 13.46) |
| Diet high in sugar-sweetened beverages | 3.92(1.91, 6.08) | 2.27(1.16, 3.37) | 4.84(2.46, 6.94) | 4.01(2.12, 5.82) |
| Diet low in fruits | 3.39(0.58, 6.03) | 2.44(0.41, 4.40) | 5.05(0.77, 8.86) | 4.48(0.70, 7.82) |
| Household air pollution from solid fuels | 2.57(0.44, 8.01) | 3.15(0.55, 9.75) | 5.42(2.75, 9.23) | 5.28(2.57, 9.30) |
| Low physical activity | 2.29(0.83, 4.26) | 6.62(2.67, 10.29) | 2.48(1.04, 3.88) | 7.34(3.15, 11.28) |
| Diet low in fiber | 0.95(0.50, 1.52) | 0.69(0.36, 1.03) | 1.09(0.60, 1.56) | 1.03(0.59, 1.47) |
| Low temperature | 0.40(0.25, 0.63) | 2.02(1.42, 2.72) | 0.74(0.45, 1.14) | 1.82(1.28, 2.50) |
| High temperature | 0.08(-0.03, 0.24) | 0.37(-0.10, 1.00) | 0.72(0.26, 1.33) | 1.25(0.42, 2.24) |
| Diet low in vegetables | 0.01(-0.00, 0.04) | 0.01(-0.00, 0.04) | 0.89(-0.35, 1.94) | 0.78(-0.29, 1.71) |

UI: Uncertainty Interval, T2D: Type 2 diabetes.

# Supplementary Table 5 Proportion of attributable risk factors for DALY burden of early-onset type 2 diabetes and T2D in China by sex, 2021

| **Attributable risk factor** | **Proportion in Both sexes, %** **(95% UI)** | | **Proportion in Men, %** **(95% UI)** | | **Proportion in Women%** **(95% UI)** | |
| --- | --- | --- | --- | --- | --- | --- |
|  | **Early-onset type 2 diabetes** | **T2D** | **Early-onset type 2 diabetes** | **T2D** | **Early-onset type 2 diabetes** | **T2D** |
| High body-mass index | 59.85(33.54, 76.65) | 51.40(24.11, 72.31) | 61.48(34.83, 78.13) | 50.04(23.74, 70.28) | 57.49(31.66, 74.06) | 52.92(24.53, 74.67) |
| Ambient particulate matter pollution | 14.77(8.24, 21.24) | 16.67(9.01, 23.93) | 15.09(8.63, 21.73) | 17.04(9.60, 24.38) | 14.32(7.49, 20.69) | 16.27(8.27, 23.54) |
| Diet high in red meat | 9.33(-1.42, 20.06) | 9.28(-1.42, 20.18) | 9.35(-1.47, 20.43) | 9.36(-1.42, 20.23) | 9.29(-1.45, 20.24) | 9.20(-1.42, 20.13) |
| Smoking | 8.94(7.59, 10.30) | 11.20(9.44, 12.83) | 14.62(12.48, 16.80) | 19.43(16.59, 22.02) | 0.73(0.44, 1.14) | 2.04(1.52, 2.56) |
| Diet low in whole grains | 7.89(2.40, 13.18) | 7.43(2.22, 12.20) | 8.31(2.55, 13.96) | 7.96(2.36, 13.28) | 7.29(2.15, 12.21) | 6.84(2.03, 11.22) |
| Secondhand smoke | 7.03(2.54, 11.45) | 7.39(2.76, 11.93) | 4.61(1.62, 7.90) | 4.90(1.72, 8.02) | 10.52(3.88, 16.68) | 10.16(3.74, 16.37) |
| Diet high in processed meat | 6.00(1.45, 10.35) | 5.04(1.22, 8.78) | 5.48(1.35, 9.75) | 4.64(1.10, 8.00) | 6.74(1.57, 11.92) | 5.49(1.35, 9.63) |
| Diet high in sugar-sweetened beverages | 3.92(1.91, 6.08) | 2.27(1.16, 3.37) | 3.62(1.63, 5.92) | 2.13(1.05, 3.24) | 4.35(1.86, 7.04) | 2.42(1.21, 3.70) |
| Diet low in fruits | 3.39(0.58, 6.03) | 2.44(0.41, 4.40) | 3.53(0.58, 6.48) | 2.61(0.43, 4.68) | 3.19(0.57, 5.96) | 2.24(0.40, 4.25) |
| Household air pollution from solid fuels | 2.57(0.44, 8.01) | 3.15(0.55, 9.75) | 2.31(0.37, 7.31) | 2.75(0.45, 8.63) | 2.94(0.53, 9.12) | 3.60(0.65, 10.87) |
| Low physical activity | 2.29(0.83, 4.26) | 6.62(2.67, 10.29) | 1.48(0.31, 3.59) | 4.81(2.02, 7.77) | 3.45(1.06, 6.89) | 8.64(3.52, 13.52) |
| Diet low in fiber | 0.95(0.50, 1.52) | 0.69(0.36, 1.03) | 0.89(0.42, 1.48) | 0.66(0.34, 1.05) | 1.04(0.53, 1.72) | 0.73(0.38, 1.11) |
| Low temperature | 0.40(0.25, 0.63) | 2.02(1.42, 2.72) | 0.49(0.30, 0.76) | 1.93(1.34, 2.68) | 0.28(0.16, 0.45) | 2.11(1.41, 2.92) |
| High temperature | 0.08(-0.03, 0.24) | 0.37(-0.10, 1.00) | 0.10(-0.04, 0.30) | 0.37(-0.10, 0.98) | 0.06(-0.02, 0.16) | 0.38(-0.09, 1.01) |
| Diet low in vegetables | 0.01(-0.00, 0.04) | 0.01(-0.00, 0.04) | 0.02(-0.00, 0.06) | 0.02(-0.01, 0.04) | 0.01(-0.00, 0.04) | 0.01(-0.00, 0.04) |

UI: Uncertainty Interval, T2D: Type 2 diabetes.

# Supplementary Table 6 Proportion of attributable risk factors for DALY burden of early-onset type 2 diabetes and T2D in Global by sex,2021

| **Attributable risk factor** | **Proportion in Both sexes, %** **(95% UI)** | | **Proportion in Men, %** **(95% UI)** | | **Proportion in Women%** **(95% UI)** | |
| --- | --- | --- | --- | --- | --- | --- |
|  | **Early-onset type 2 diabetes** | **T2D** | **Early-onset type 2 diabetes** | **T2D** | **Early-onset type 2 diabetes** | **T2D** |
| High body-mass index | 57.21(32.47, 72.69) | 52.15(24.82, 71.80) | 56.70(32.14, 72.30) | 50.33(23.82, 69.69) | 57.81(32.95, 73.13) | 54.03(25.84, 74.02) |
| Ambient particulate matter pollution | 10.04(5.81, 14.76) | 11.83(6.77, 17.36) | 10.71(6.31, 15.78) | 12.19(6.95, 17.88) | 9.27(5.26, 13.66) | 11.46(6.52, 16.78) |
| Diet high in processed meat | 7.03(1.69, 11.62) | 8.08(1.96, 13.46) | 6.70(1.61, 11.12) | 7.87(1.91, 13.11) | 7.41(1.79, 12.31) | 8.29(2.01, 13.81) |
| Diet low in whole grains | 6.72(1.93, 10.84) | 6.73(1.95, 11.02) | 7.08(2.04, 11.50) | 7.13(2.07, 11.71) | 6.30(1.81, 10.18) | 6.31(1.83, 10.28) |
| Smoking | 5.66(4.83, 6.53) | 7.25(6.06, 8.54) | 9.31(7.98, 10.77) | 11.84(9.93, 13.82) | 1.45(1.18, 1.71) | 2.54(2.09, 3.08) |
| Household air pollution from solid fuels | 5.42(2.75, 9.23) | 5.28(2.57, 9.30) | 4.99(2.49, 8.80) | 4.87(2.39, 8.76) | 5.92(3.04, 9.84) | 5.70(2.75, 9.97) |
| Diet low in fruits | 5.05(0.77, 8.86) | 4.48(0.70, 7.82) | 5.07(0.76, 8.85) | 4.46(0.69, 7.77) | 5.01(0.78, 8.77) | 4.50(0.71, 7.88) |
| Diet high in sugar-sweetened beverages | 4.84(2.46, 6.94) | 4.01(2.12, 5.82) | 4.62(2.40, 6.70) | 3.88(2.05, 5.63) | 5.10(2.57, 7.43) | 4.15(2.18, 6.04) |
| Secondhand smoke | 4.78(1.77, 7.90) | 4.89(1.77, 8.03) | 3.93(1.42, 6.51) | 4.09(1.46, 6.77) | 5.77(2.14, 9.36) | 5.71(2.08, 9.37) |
| Diet high in red meat | 4.56(-0.66, 10.01) | 5.06(-0.74, 11.32) | 4.74(-0.69, 10.49) | 5.17(-0.76, 11.51) | 4.36(-0.66, 9.58) | 4.94(-0.73, 11.05) |
| Low physical activity | 2.48(1.04, 3.88) | 7.34(3.15, 11.28) | 1.69(0.73, 2.67) | 5.49(2.31, 8.45) | 3.39(1.36, 5.29) | 9.23(4.02, 13.98) |
| Diet low in fiber | 1.09(0.60, 1.56) | 1.03(0.59, 1.47) | 1.07(0.60, 1.53) | 0.99(0.57, 1.41) | 1.12(0.63, 1.59) | 1.06(0.61, 1.52) |
| Diet low in vegetables | 0.89(-0.35, 1.94) | 0.78(-0.29, 1.71) | 0.87(-0.34, 1.88) | 0.78(-0.28, 1.67) | 0.92(-0.36, 2.00) | 0.79(-0.30, 1.75) |
| Low temperature | 0.74(0.45, 1.14) | 1.82(1.28, 2.50) | 0.73(0.46, 1.11) | 1.81(1.28, 2.48) | 0.74(0.43, 1.16) | 1.84(1.26, 2.56) |
| High temperature | 0.72(0.26, 1.33) | 1.25(0.42, 2.24) | 0.66(0.24, 1.25) | 1.22(0.42, 2.29) | 0.79(0.28, 1.46) | 1.27(0.42, 2.26) |

UI: Uncertainty Interval, T2D: Type 2 diabetes.

# Supplementary Table 7 Temporal trends for the proportion of the top five attributable risk factors of DALYs for early-onset type 2 diabetes in China from 1990 to 2021

| year | High body-mass index | Ambient particulate matter pollution | Diet high in red meat | Smoking | Diet low in whole grains |
| --- | --- | --- | --- | --- | --- |
| 1990 | 40.08(20.71, 55.79) | 3.46(1.53, 6.28) | 4.94(-0.71, 11.65) | 9.38(7.88, 10.92) | 6.42(1.79, 10.55) |
| 1991 | 40.66(21.03, 56.39) | 3.59(1.69, 6.41) | 5.04(-0.73, 11.86) | 9.39(7.89, 10.94) | 6.62(1.85, 10.93) |
| 1992 | 41.24(21.38, 57.15) | 3.73(1.87, 6.43) | 5.14(-0.75, 12.00) | 9.41(7.95, 10.96) | 6.82(1.90, 11.36) |
| 1993 | 41.80(21.73, 57.79) | 3.88(1.95, 6.59) | 5.25(-0.78, 12.30) | 9.43(8.00, 10.93) | 6.99(1.97, 11.64) |
| 1994 | 42.31(21.95, 58.34) | 4.04(1.99, 7.00) | 5.37(-0.79, 12.50) | 9.43(7.99, 10.96) | 7.15(2.01, 11.92) |
| 1995 | 42.81(22.19, 58.93) | 4.22(1.93, 7.66) | 5.51(-0.82, 12.70) | 9.46(8.02, 11.02) | 7.31(2.05, 12.18) |
| 1996 | 43.29(22.55, 59.36) | 4.40(2.15, 7.60) | 5.63(-0.85, 12.88) | 9.37(8.00, 10.79) | 7.45(2.10, 12.44) |
| 1997 | 43.76(22.86, 59.88) | 4.58(2.35, 7.63) | 5.75(-0.87, 13.12) | 9.19(7.85, 10.60) | 7.58(2.14, 12.64) |
| 1998 | 44.30(23.18, 60.38) | 4.80(2.34, 8.14) | 5.88(-0.88, 13.39) | 9.05(7.78, 10.43) | 7.71(2.18, 12.84) |
| 1999 | 44.87(23.52, 60.85) | 5.05(2.38, 8.49) | 6.03(-0.90, 13.66) | 9.01(7.73, 10.34) | 7.83(2.22, 13.01) |
| 2000 | 45.45(23.81, 61.47) | 5.33(2.51, 9.02) | 6.18(-0.92, 13.93) | 9.09(7.79, 10.44) | 7.93(2.24, 13.10) |
| 2001 | 46.02(24.29, 61.99) | 5.65(2.72, 9.21) | 6.33(-0.95, 14.20) | 9.19(7.86, 10.53) | 8.00(2.26, 13.16) |
| 2002 | 46.56(24.68, 62.46) | 6.00(2.98, 9.86) | 6.48(-0.97, 14.52) | 9.25(7.93, 10.58) | 8.04(2.26, 13.19) |
| 2003 | 47.08(25.11, 62.92) | 6.37(3.23, 10.32) | 6.63(-0.99, 14.79) | 9.25(7.92, 10.60) | 8.05(2.26, 13.18) |
| 2004 | 47.61(25.50, 63.33) | 6.76(3.35, 10.82) | 6.78(-1.01, 15.10) | 9.18(7.88, 10.55) | 8.04(2.25, 13.12) |
| 2005 | 48.14(25.82, 63.91) | 7.14(3.48, 11.47) | 6.92(-1.03, 15.40) | 9.07(7.72, 10.40) | 7.99(2.21, 13.01) |
| 2006 | 48.73(26.30, 64.49) | 7.54(3.74, 11.81) | 7.05(-1.06, 15.79) | 8.93(7.57, 10.26) | 7.91(2.19, 12.86) |
| 2007 | 49.38(26.79, 65.24) | 8.00(3.99, 12.50) | 7.20(-1.09, 16.10) | 8.81(7.50, 10.16) | 7.82(2.16, 12.71) |
| 2008 | 50.08(27.28, 66.02) | 8.50(4.30, 13.21) | 7.35(-1.11, 16.38) | 8.66(7.34, 9.99) | 7.74(2.14, 12.56) |
| 2009 | 50.80(27.73, 66.87) | 9.03(4.57, 14.00) | 7.52(-1.14, 16.62) | 8.50(7.20, 9.82) | 7.70(2.13, 12.49) |
| 2010 | 51.53(28.15, 67.77) | 9.57(4.79, 14.73) | 7.71(-1.15, 16.98) | 8.31(7.03, 9.55) | 7.69(2.15, 12.52) |
| 2011 | 52.25(28.69, 68.53) | 10.21(5.20, 15.52) | 7.92(-1.19, 17.43) | 8.08(6.85, 9.30) | 7.71(2.17, 12.54) |
| 2012 | 53.00(29.27, 69.45) | 10.98(5.70, 16.47) | 8.14(-1.24, 17.88) | 7.87(6.72, 9.06) | 7.77(2.20, 12.62) |
| 2013 | 53.77(29.75, 70.27) | 11.80(6.20, 17.53) | 8.35(-1.29, 18.28) | 7.75(6.61, 8.92) | 7.84(2.24, 12.71) |
| 2014 | 54.58(30.24, 71.15) | 12.58(6.69, 18.51) | 8.57(-1.32, 18.71) | 7.75(6.59, 8.98) | 7.93(2.29, 12.86) |
| 2015 | 55.38(30.67, 72.10) | 13.19(7.11, 19.36) | 8.77(-1.35, 19.20) | 7.81(6.67, 9.03) | 8.00(2.33, 13.04) |
| 2016 | 56.15(31.15, 72.90) | 13.65(7.40, 19.87) | 8.94(-1.38, 19.55) | 7.94(6.78, 9.15) | 8.04(2.35, 13.10) |
| 2017 | 56.88(31.58, 73.70) | 14.05(7.65, 20.27) | 9.08(-1.42, 19.85) | 8.13(6.96, 9.37) | 8.04(2.36, 13.17) |
| 2018 | 57.61(32.06, 74.41) | 14.38(7.91, 20.73) | 9.20(-1.44, 20.09) | 8.34(7.12, 9.68) | 8.01(2.37, 13.21) |
| 2019 | 58.34(32.53, 75.20) | 14.64(8.08, 21.08) | 9.29(-1.44, 20.24) | 8.55(7.31, 9.91) | 7.97(2.39, 13.15) |
| 2020 | 59.16(33.07, 75.84) | 14.83(8.30, 21.34) | 9.33(-1.43, 20.13) | 8.77(7.47, 10.10) | 7.93(2.39, 13.25) |
| 2021 | 59.85(33.54, 76.65) | 14.77(8.24, 21.24) | 9.33(-1.42, 20.06) | 8.94(7.59, 10.30) | 7.89(2.40, 13.18) |

# Supplementary Table 8 Temporal trends for the proportion of the top five attributable risk factors of DALYs for early-onset type 2 diabetes in Global from 1990 to 2021

| year | High body-mass index | Ambient particulate matter pollution | Diet high in processed meat | Diet low in whole grains | Smoking |
| --- | --- | --- | --- | --- | --- |
| 1990 | 43.61(23.13, 58.18) | 5.96(3.45, 8.82) | 5.65(1.31, 9.32) | 6.01(1.69, 9.72) | 7.53(6.41, 8.65) |
| 1991 | 44.09(23.44, 58.65) | 6.08(3.49, 8.87) | 5.72(1.33, 9.43) | 6.06(1.70, 9.79) | 7.46(6.34, 8.57) |
| 1992 | 44.56(23.74, 59.14) | 6.18(3.58, 8.95) | 5.77(1.34, 9.54) | 6.09(1.71, 9.84) | 7.38(6.30, 8.49) |
| 1993 | 45.01(24.06, 59.77) | 6.29(3.68, 9.14) | 5.82(1.36, 9.62) | 6.14(1.73, 9.88) | 7.32(6.27, 8.40) |
| 1994 | 45.47(24.30, 60.25) | 6.39(3.75, 9.31) | 5.87(1.37, 9.69) | 6.17(1.74, 9.98) | 7.25(6.20, 8.31) |
| 1995 | 45.85(24.53, 60.72) | 6.48(3.82, 9.46) | 5.89(1.38, 9.69) | 6.20(1.75, 10.06) | 7.19(6.17, 8.27) |
| 1996 | 46.16(24.76, 61.05) | 6.53(3.86, 9.55) | 5.87(1.38, 9.68) | 6.24(1.76, 10.11) | 7.12(6.10, 8.21) |
| 1997 | 46.45(24.97, 61.40) | 6.55(3.88, 9.59) | 5.81(1.37, 9.53) | 6.32(1.78, 10.24) | 7.05(6.02, 8.11) |
| 1998 | 46.74(25.17, 61.70) | 6.58(3.91, 9.64) | 5.72(1.35, 9.45) | 6.41(1.81, 10.40) | 7.01(6.00, 8.06) |
| 1999 | 47.09(25.41, 62.08) | 6.66(3.96, 9.85) | 5.65(1.33, 9.35) | 6.48(1.83, 10.54) | 6.97(5.98, 8.00) |
| 2000 | 47.50(25.63, 62.55) | 6.74(4.01, 10.05) | 5.63(1.33, 9.33) | 6.53(1.84, 10.58) | 6.95(5.94, 7.98) |
| 2001 | 47.96(26.02, 63.13) | 6.86(4.10, 10.12) | 5.68(1.34, 9.42) | 6.56(1.85, 10.62) | 6.91(5.90, 7.92) |
| 2002 | 48.47(26.41, 63.63) | 6.99(4.18, 10.34) | 5.75(1.35, 9.54) | 6.58(1.85, 10.62) | 6.84(5.85, 7.83) |
| 2003 | 48.96(26.78, 64.15) | 7.12(4.25, 10.46) | 5.83(1.38, 9.65) | 6.59(1.85, 10.61) | 6.75(5.74, 7.75) |
| 2004 | 49.41(27.09, 64.60) | 7.23(4.31, 10.70) | 5.91(1.40, 9.81) | 6.60(1.85, 10.61) | 6.65(5.65, 7.61) |
| 2005 | 49.86(27.36, 65.11) | 7.35(4.35, 10.96) | 6.02(1.42, 9.98) | 6.60(1.85, 10.61) | 6.53(5.55, 7.49) |
| 2006 | 50.31(27.68, 65.55) | 7.47(4.41, 11.10) | 6.11(1.45, 10.15) | 6.59(1.84, 10.55) | 6.42(5.46, 7.36) |
| 2007 | 50.76(28.04, 66.00) | 7.60(4.47, 11.26) | 6.20(1.47, 10.31) | 6.58(1.84, 10.53) | 6.32(5.39, 7.26) |
| 2008 | 51.22(28.35, 66.49) | 7.73(4.56, 11.48) | 6.27(1.49, 10.45) | 6.57(1.84, 10.52) | 6.20(5.29, 7.10) |
| 2009 | 51.73(28.72, 67.05) | 7.89(4.64, 11.70) | 6.36(1.52, 10.61) | 6.57(1.84, 10.51) | 6.08(5.16, 6.98) |
| 2010 | 52.18(28.98, 67.56) | 8.04(4.70, 11.91) | 6.45(1.54, 10.73) | 6.59(1.85, 10.56) | 5.96(5.07, 6.85) |
| 2011 | 52.61(29.33, 68.01) | 8.28(4.83, 12.25) | 6.54(1.56, 10.89) | 6.61(1.85, 10.63) | 5.84(4.95, 6.72) |
| 2012 | 53.10(29.68, 68.50) | 8.64(5.06, 12.78) | 6.64(1.59, 11.02) | 6.64(1.86, 10.69) | 5.72(4.86, 6.59) |
| 2013 | 53.59(30.02, 69.03) | 9.05(5.30, 13.25) | 6.71(1.61, 11.17) | 6.66(1.87, 10.74) | 5.61(4.79, 6.48) |
| 2014 | 54.11(30.37, 69.55) | 9.42(5.53, 13.71) | 6.80(1.64, 11.31) | 6.69(1.88, 10.79) | 5.56(4.75, 6.42) |
| 2015 | 54.60(30.62, 70.01) | 9.66(5.65, 14.05) | 6.88(1.66, 11.46) | 6.72(1.89, 10.82) | 5.53(4.71, 6.39) |
| 2016 | 55.07(30.97, 70.46) | 9.79(5.72, 14.28) | 6.95(1.68, 11.55) | 6.73(1.90, 10.87) | 5.51(4.71, 6.37) |
| 2017 | 55.52(31.35, 70.97) | 9.88(5.76, 14.47) | 7.01(1.69, 11.64) | 6.74(1.90, 10.87) | 5.53(4.71, 6.40) |
| 2018 | 56.02(31.68, 71.47) | 9.96(5.78, 14.60) | 7.07(1.70, 11.71) | 6.75(1.91, 10.88) | 5.56(4.73, 6.42) |
| 2019 | 56.45(31.93, 71.93) | 10.01(5.79, 14.68) | 7.10(1.71, 11.77) | 6.74(1.92, 10.88) | 5.59(4.75, 6.46) |
| 2020 | 56.85(32.17, 72.26) | 10.00(5.74, 14.65) | 7.05(1.70, 11.67) | 6.71(1.92, 10.80) | 5.65(4.80, 6.53) |
| 2021 | 57.21(32.47, 72.69) | 10.04(5.81, 14.76) | 7.03(1.69, 11.62) | 6.72(1.93, 10.84) | 5.66(4.83, 6.53) |
